# Efficacy and tolerability Aripiprazole once-monthly long-acting injectable in schizophrenia. Two-injection start regimen. A 24 months follow-up and mirror image study

**DOI:** 10.1192/j.eurpsy.2024.1564

**Published:** 2024-08-27

**Authors:** S. L. Romero Guillena, F. Gotor Sanchez-Luengo, B. O. Plasencia Garcia de Diego

**Affiliations:** ^1^Psychiatry, UGC Mental Health Virgen Macarena; ^2^Psychiatry, UGC Mental Heaslth Virgen Rocio; ^3^Psychiatry, UGC Mental Health Virgen Rocio, Seville, Spain

## Abstract

**Introduction:**

Relapse prevention is crucial in patients with schizophrenia, as repeated episodes can worsen psychopathology and functionality. There is strong evidence of antipsychotics efficacy in preventing relapse; however, non-compliance rates in patients with schizophrenia are very high. Long-acting injectable antipsychotics (LAIs) are an important treatment option but remain underutilized.

Aripiprazole once-monthly is a long-acting intramuscular injectable formulation of aripiprazole indicated for the maintenance treatment of schizophrenia in adult patients stabilized on oral aripiprazole.

If one injection start regimen is adopted, on the day of initiation, an injection of 400mg Aripiprazole once monthly should be administered accompanied by 10mg to 20mg of oral aripiprazole per day for the successive 14 days New treatment regimen: On the day it begins, inject 400 mg Aripiprazole twice at different sites and provide one 20 mg dose of oral aripiprazole

**Objectives:**

The main aim of this study is to evaluate the efficacy and tolerance of Aripiprazole long-acting injectable (ALAI) in stable patients with schizophrenia.The initial dose was administered according to the new regimen (Two injection Start).

The secondary objective is to compare hospitalizations and emergency interventions during 24 months before (retrospective) and after (prospective) switching to ALAI.

**Methods:**

The study included 15 patients diagnosed with stable schizophrenia (DSM 5 criteria) who underwent treatment with ALAI. The beginning dosage was administered using the new regimen (Two Injection Start).

Over an 24-month follow-up period, the Clinical Global Impression-Schizophrenia scale (CGI-SCH), treatment adherence, concomitant medication, hospitalizations, emergency assists, and reported side effects were evaluated every three months.

**Results:**

Mean initial scores were 4.24 (±0.83) on GCI-SCH.

After 24 months, the mean scores varied from baseline by -1.21±0.74 (P<0.01) on the ICG-SCH.

The percentage of patients who remained admission-free at the end of the 24 months was 73%.

The treatment adherence rate for ALAI after 24 months was 66%.

The most frequent side effect with an incidence of 20% was transient mild insomnia. None of the patients who started ALAI after the 2-injection start regimen experienced severe adverse effects or severe adverse effects.

There were 20 hospital admissions during the 24-month period prior to the switch to ALI, which fell to 5 hospital admissions 24 months following the switch.

Similarly, there were 38 emergency assists during the 24-month period before the switch to ALI, which dropped to 9 emergency assists 24 months after the switch.

**Conclusions:**

We found of Aripiprazole long-acting injectable (The starting dose was administered following the new regimen (Two injection Start)) is effective, safe, and well tolerated in clinical practice conditions

**Disclosure of Interest:**

None Declared

